# A Fusion Algorithm Based on a Constant Velocity Model for Improving the Measurement of Saccade Parameters with Electrooculography

**DOI:** 10.3390/s24020540

**Published:** 2024-01-15

**Authors:** Palpolage Don Shehan Hiroshan Gunawardane, Raymond Robert MacNeil, Leo Zhao, James Theodore Enns, Clarence Wilfred de Silva, Mu Chiao

**Affiliations:** 1 Department of Mechanical Engineering, The University of British Columbia, Vancouver, BC V6T 1Z4, Canada; hiroshan@mail.ubc.ca (P.D.S.H.G.); leozhao1@hotmail.com (L.Z.); desilva@mech.ubc.ca (C.W.d.S.); 2 Department of Psychology, The University of British Columbia, Vancouver, BC V6T 1Z4, Canada; raymond.macneil@psych.ubc.ca (R.R.M.); muchiao@mech.ubc.ca (M.C.)

**Keywords:** biomedical signal processing, corneo-retinal potential, electrooculography, filtering algorithms, eye tracking, Kalman filters, saccades

## Abstract

Electrooculography (EOG) serves as a widely employed technique for tracking saccadic eye movements in a diverse array of applications. These encompass the identification of various medical conditions and the development of interfaces facilitating human–computer interaction. Nonetheless, EOG signals are often met with skepticism due to the presence of multiple sources of noise interference. These sources include electroencephalography, electromyography linked to facial and extraocular muscle activity, electrical noise, signal artifacts, skin-electrode drifts, impedance fluctuations over time, and a host of associated challenges. Traditional methods of addressing these issues, such as bandpass filtering, have been frequently utilized to overcome these challenges but have the associated drawback of altering the inherent characteristics of EOG signals, encompassing their shape, magnitude, peak velocity, and duration, all of which are pivotal parameters in research studies. In prior work, several model-based adaptive denoising strategies have been introduced, incorporating mechanical and electrical model-based state estimators. However, these approaches are really complex and rely on brain and neural control models that have difficulty processing EOG signals in real time. In this present investigation, we introduce a real-time denoising method grounded in a constant velocity model, adopting a physics-based model-oriented approach. This approach is underpinned by the assumption that there exists a consistent rate of change in the cornea-retinal potential during saccadic movements. Empirical findings reveal that this approach remarkably preserves EOG saccade signals, resulting in a substantial enhancement of up to 29% in signal preservation during the denoising process when compared to alternative techniques, such as bandpass filters, constant acceleration models, and model-based fusion methods.

## 1. Introduction

Ocular motion sensing, also referred to as eye tracking, is utilized in both medical and engineering applications [1,2,3,4,5]. These systems leverage various types of eye movements, encompassing saccades, smooth pursuit, vergences, and vestibular–ocular reflexes [6,7]. Among the array of technologies available for sensing eye movement, electrooculography (EOG) and videooculography (VOG) stand as the predominant techniques [8]. EOG relies on the deployment of electrodes to measure the relative changes in the cornea-retinal potential that manifest during the simultaneous rotation of the eye and the cornea-retinal potential’s field vector [9]. On the other hand, VOG harnesses the infrared signals’ reflection on the corneal–retinal surface to gauge the eye’s angular displacement [8]. VOG systems are equipped with high-speed video cameras, surpassing EOG systems in accuracy. Notably, commercially available VOG systems, such as the EyeLink 1000 eye-tracker (EL), possess the capability to capture eye movements at a remarkable sampling rate of 2000 Hz , with an accuracy reaching an impressive 0.001 degrees of visual angle (DVA) [10]. However, it is essential to recognize that VOG systems do entail several drawbacks. They tend to be bulky, come with substantial costs, necessitate stringent or controlled lighting conditions, and fall short in their capacity to monitor closed-eye movements. These inherent limitations render EOG systems an indispensable choice for tracking eye movements across diverse scenarios where the use of VOG systems may be unfeasible. Notably, in contexts such as sleep disorder monitoring, EOG becomes indispensable, enabling the measurement of eye movements even when the eyes are closed [2,11].

Among the different types of eye movements, saccades hold paramount significance in medical and engineering applications. A saccade is defined as a swift and ballistic eye motion that redirects the point of fixation from one relatively stable position to another [5]. Both EOG and VOG systems possess the capability to capture this eye movement. Nevertheless, EOG systems suffer from a notable drawback—the presence of a substantial volume of noise and artifacts that detrimentally impact the fidelity of the recorded EOG eye movements. A typical EOG signal exhibits the intrusion of diverse artifacts, including but not limited to electroencephalography (EEG) artifacts, electromyography (EMG) artifacts, blink artifacts, and an assortment of noise sources [12,13]. These artifacts coexist within similar frequency spectra, complicating the denoising procedure [14,15]. Consequently, the extraction of saccades and the associated attributes, such as saccade amplitude, velocity, and latency [5,10], from an EOG recording becomes an onerous and time-intensive endeavor [16,17]. This onerous process exerts a detrimental influence on the accurate identification and classification of saccades [14].

Contemporary traditional saccade detection and classification methods, which rely on current threshold-based techniques, necessitate EOG signals characterized by minimal noise levels to effectively discern abrupt changes in amplitude and velocity profiles [18]. To attain this objective, conventional signal processing techniques such as bandpass filters [18,19], wavelet transforms [20,21,22], and smoothing filters [14,15], as well as specialized filters like morphological filters [23] and dynamic time-warping filters [24], have been employed to denoise EOG signals. Nonetheless, these traditional filtering methods often introduce distortions by inadvertently diminishing peak velocities and extending saccade duration during the denoising process [14,15,18]. Furthermore, they tend to compromise the preservation of EOG saccades and result in a significant deviation from the ground truth [10]. In response to these limitations, adaptive filtering approaches have been developed to effectively filter EOG signals while retaining the fidelity of EOG saccades [25]. Notably, Kalman filters (KFs) [26] have been introduced as a means to fuse the measured EOG signals with values estimated using a predefined mathematical model [26,27,28]. This approach has shown substantial improvements in maintaining the shape and integrity of EOG saccades [10]. Comparative studies have corroborated the superiority of KFs over traditional filtering methods in denoising EOG signals [25]. Nevertheless, it is worth noting that the accuracy of a KF is contingent on the precision of the underlying state estimator [10].

Diverse KF state estimators have been developed, drawing upon the mechanical [10,20,29], electrical [30,31], and parametric [15,32] attributes of the eye and its ocular movements. Notably, lumped-element-based dynamic models have served as foundational state estimators in this context [10,20,29]. Within these state estimators, the intricate agonist–antagonist dynamics of the extraocular muscles are characterized by their electromechanical properties to accurately estimate saccades, and these estimations are effectively integrated with EOG signals, leading to substantial enhancements in EOG saccade fidelity [10].

While most of the model-based techniques have demonstrated proficiency in addressing issues such as the elimination of eye blinks, offset correction, and signal denoising, they have typically necessitated real-time operation facilitated by a brain or neural controller [10,29]. Similarly, electrical models rooted in Coulomb’s law have been developed to rectify the baseline drift in EOG signals [25,30], but they encounter comparable challenges in preserving the integrity of EOG saccades during the denoising process. In VOG systems, KF fusion-based methodologies have been employed to denoise ocular motion signals derived from pupil reflections [33]. Nevertheless, these model-based denoising approaches [34,35], which utilize acceleration-based fusion algorithms [25], exhibit a notable disparity in accuracy when compared to ground truth measurements.

The present study explores a novel model-based technique aimed at enhancing the denoising of EOG signals while concurrently preserving the fidelity of EOG saccades. Specifically, we introduce a constant velocity-based model that considers the relationship between the peak velocity and saccade amplitude in the human eye, serving as a state estimator for the KF. We evaluate the effectiveness of this model in retaining EOG saccades throughout the denoising process and compare its performance to several traditional and adaptive denoising methods. This paper is structured as follows: Section 2 provides the mathematical foundation of the model-based fusion algorithms, experimental procedures employed for data acquisition and the algorithmic techniques employed for saccade identification and measurement. Section 3 and Section 4 present the outcomes of our analyses and the ensuing conclusions, respectively.

## 2. Materials and Methods

In this section, we introduce the constant velocity-based (CVM) KF method, where $\frac{dE_{e}}{dt}$ is held at a constant value, and we present other established techniques for performance comparison. Well-established KF-related approaches, including bandpass filters (BP) [18,19], Brownian model-based (BM) KFs [17], constant acceleration model-based (CAM) KFs [25] (where $\frac{d^{2}E_{e}}{dt^{2}}$ is kept constant), and linear reciprocal (LR) model-based filters [10], are employed as benchmarks to assess and evaluate the efficacy of the CVM.

### 2.1. Theoretical Models

#### 2.1.1. Saccadic Eye Movements and EOG Signals

EOG is a method for measuring the corneal–retinal potential of the eye. This potential exhibits linearity with regard to angular displacements, spanning from −35 ° to 35 ° for horizontal movements and −10 ° to 10 ° for vertical movements [1,9]. The amplitude of EOG signals ranges from 50 to 3500 μ V for both horizontal and vertical gaze movements and falls within the frequency band of 1 to 35 Hz . Notably, the voltage sensitivity to changes during horizontal and vertical movements (saccades) is approximately 16 μ V and 14 μ V DVA, respectively [1,9]. In the design of adaptive filters, effective noise models are crucial, and the modeling and analysis of noise in biomedical systems have been elaborated upon in a previous work [36]. For the purposes of this study, an additive white noise model is employed to represent the noise inherent in EOG signals. This assumption has been effectively utilized by previous researchers in the development of their denoising methods [10,20,32].

A raw EOG signal comprises a combination of the corneo-retinal potential of the eye, noise, and artifacts. The induced voltage in the EOG electrode, denoted as E(t) , can be described as follows:

(1) $$E\left(t\right)=E_{e}\left(t\right)+\varphi +\Omega$$

where $E_{e}\left(t\right)$ = corneal–retinal potential, φ = artifacts (eye muscles, eyelids, blinks, etc.), and Ω = noise (electromechanical noise). However, $E_{e}\left(t\right)$ is linearly proportional to the rotation of the eyeball. Therefore,

(2) $$E_{e}\left(t\right)=K\theta_{axis}\left(t\right)$$

where $\theta_{axis}\left(t\right)$ is the angular displacement around the eyeball axis parallel to the location of the electrode, and *K* is the calibration factor [10].

#### 2.1.2. Noise Model

**Figure 1:**
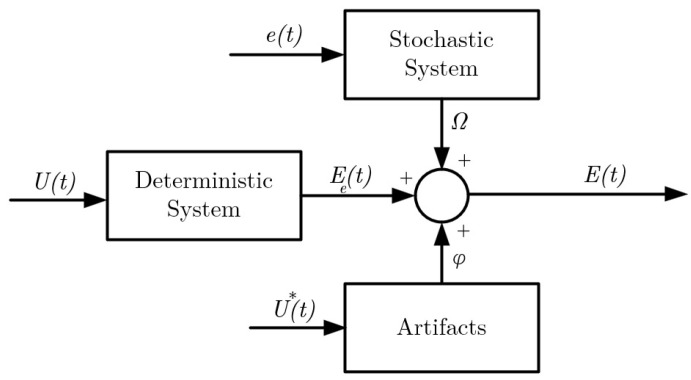
Noise model for a raw EOG signal. U(t) : input to the deterministic systems (visual cue), $U^{*}\left(t\right)$ : trigger signal for the artifacts, $E_{e}\left(t\right)$ : output of the deterministic signal (corneo-retinal potential), e(t) : white noise signal to the stochastic system, Ω : additive noise signal, E(t) : raw EOG signal, and φ : artifacts [10].

The noise inherent in EOG can be effectively characterized, as illustrated in Figure 1, with reference to prior studies [10,20,32,36]. In this depiction, U(t) serves as the input to the deterministic plant, providing the visual cues necessary to initiate saccades, resulting in the generation of an output denoted as $E_{e}\left(t\right)$ . A noise signal e(t) introduces stochasticity to the process and gives rise to the additive noise component Ω in the signal. Additionally, $U^{*}\left(t\right)$ is responsible for triggering artifacts, such as blinks, in the system. The ultimate measurement received by the electrode, E(t) , represents a composite of all these contributing factors and artifacts. The accurate identification of the deterministic system is of paramount importance to mitigate the adverse effects of the stochastic component. In this context, a variety of corneal–retinal potential models, serving as state estimators, are explored within the framework of this model (as discussed in Section 2.1.3). In the present study, the stochastic system is described using a Gaussian noise model, while the deterministic system is represented by the CVM.

#### 2.1.3. Corneo-Retinal Potential Models for Saccades

The sensor fusion approach introduced in this paper is based on a KF, which employs linear quadratic estimation to filter a sequence of measurements collected over time. In this context, both process uncertainties and uncertainties in the measured values are treated as sources of noise. The process can be mathematically represented in a discretized form as follows:

(3) $$E_{e\_k}=AE_{e\_(k-1)}+BU_{k}+\Omega_{k}$$

In this context, $E_{e\_k}$ corresponds to the discretized states of the system, which can be expressed as a 3 × 1 column vector. Here, $s_{k}$ , $v_{k}$ , and $a_{k}$ denote the biosignal, its rate of change, and the second rate of change, respectively, for the $k^{th}$ sample. The process noise, denoted as $\Omega_{k}$ , is assumed to follow an additive Gaussian white noise distribution with a covariance matrix *Q*, i.e., $\Omega_{k}∼N\left(0,Q\right)$ . Additionally, the state transition matrix, denoted as *A*, characterizes the dynamics of the system, reflecting the state transition relation. The specific form of this matrix is contingent on the time-domain process model of the system. For this study, three saccadic eye models are considered: Brownian, constant velocity, and constant acceleration. Notably, the input gain matrix *B* is omitted, as this work operates as a “free” model and does not involve an input variable $U_{k}\prime \prime$ . The measurement, denoted as $E_{e\_k}$ (EOG), is subject to measurement noise, which is assumed to follow an additive white Gaussian noise distribution with a covariance matrix *R*, i.e., $\epsilon_{k}∼N\left(0,R\right)$ . This assumption is made under the premise of no correlation between $\Omega_{k}$ and $\epsilon_{k}\prime \prime$ . The output model is represented as follows:

(4) $$E_{k}=HE_{e\_k}+\epsilon_{k}$$

where *H* represents the measurement matrix. The CVM is employed to describe $E_{e}\left(t\right)$ , which is discretized as $E_{e\_k}$ . Subsequently, for the purpose of assessing the performance of the CVM in conjunction with state-of-the-art KFs, *H* is substituted with the BM, CAM, and LR, as elucidated in subsection D.

#### Constant Velocity Model

The analysis of the peak velocity in saccades has been a common practice in studies related to ocular motion [15,32]. Controlled experiments involving saccades have revealed that the peak velocity conforms to a nonlinear function characterized by its amplitude, as depicted in Equation (5). Furthermore, the product of the peak velocity and saccade duration, as described by Equation (6), exhibits a linear correlation with the amplitude [32].

(5) $$V_{p}=a_{1}\left(1-e^{-\theta /a_{2}}\right)$$

(6) $$V_{p}\times D=\theta$$

Here, in Equations (5) and (6), $V_{p}$ denotes the peak velocity, $a_{1}$ and $a_{2}$ represent arbitrary constants, *D* signifies the duration of the saccade, and θ stands for the amplitude of the saccade. Drawing upon the findings presented in [17,32], Equation (6) can be construed as a representation of the saccade amplitude achieved by maintaining a constant velocity for a given duration. Based on this observation, we propose a linear constant velocity state estimator model for the Kalman filter (KF), under the assumption that the peak velocity remains approximately constant for higher saccade amplitudes. This assumption further implies that $\frac{dE_{e}}{dt}$ , representing the peak rate of change of the differential potential, remains relatively constant, as we are linearly associating changes in potential with the saccade amplitude. The state-space representation of this corresponding process model, in discrete form, can be expressed as follows:

(7) $$E_{e\_(k+1)}=AE_{e\_k}+K{\dot{E}}_{e\_k}$$

(8) $${\dot{E}}_{e\_(k+1)}={\dot{E}}_{e\_k}$$

Therefore, the state-space form of the model is

(9) $$\left[\begin{array}{c} E_{e\_(k+1)}\\ {\dot{E}}_{e\_(k+1)} \end{array}\right]=\left[\begin{array}{cc} 1 & k\\ 0 & 1 \end{array}\right]\left[\begin{array}{c} E_{e\_(k)}\\ {\dot{E}}_{e\_(k)} \end{array}\right]+Noise$$

#### Kalman Filter Model

The state estimation uses the following state estimation scheme of prediction and correction.

Prediction:

$${\hat{E}}_{e\_k}^{-}={\hat{E}}_{e\_(k-1)}^{-}+BU_{k}+R\;P_{k}^{-}=AP_{k-1}^{-}A^{T}+Q$$

Correction:

$$K_{k}=P_{k}^{-}H^{T}{\left(HP_{k}^{-}H^{T}+R\right)}^{-1}\;P_{k}=\left(I-K_{k}H\right)P_{k}^{-}\;{\hat{E}}_{e\_k}={\hat{E}}_{k}^{-}+K_{k}\left(Z_{k}-H{\hat{E}}_{e\_k}^{-}\right)$$

where ${\hat{E}}_{e\_k}$ = estimate, $P_{k}$ = covariance, and $K_{k}$ = Kalman gain.

#### 2.1.4. Other State Estimator Models

#### Brownian Motion

In the context of the BM model, Brownian motion serves as the foundational concept from which the state estimator is derived, as referenced in [10,17]. The state-space representation of the corresponding process model, when expressed discretely, can be written as follows:

(10) $$E_{e\_(k+1)}=AE_{e\_k}+Noise$$

where *A* = system matrix, which is an identity matrix, in the present work.

#### Constant Acceleration Model

The CAM considers ( $\frac{d^{2}E_{e}}{dt^{2}}$ ), the 2nd rate of change of the differential potential, to be a constant [10,17,25]. The state-space representation of the corresponding process model, in the discrete form, can be written as

(11) $$E_{e\_(k+1)}=E_{e\_k}+k{\dot{E}}_{e\_k}+\frac{1}{2}k^{2}{\ddot{E}}_{e\_k}$$

(12) $${\dot{E}}_{e\_(k+1)}={\dot{E}}_{e\_k}+k{\ddot{E}}_{e\_k}$$

(13) $${\dddot{E}}_{e\_(k+1)}={\ddot{E}}_{e\_k}$$

Therefore, the state-space form of the model is

(14) $$\left[\begin{array}{c} E_{e\_(k+1)}\\ {\ddot{E}}_{e\_(k+1)}\\ {\dddot{E}}_{e\_(k+1)} \end{array}\right]=\left[\begin{array}{ccc} 1 & k & \frac{1}{2}k^{2}\\ 0 & 1 & k\\ 0 & 0 & 1 \end{array}\right]\left[\begin{array}{c} E_{e\_(k)}\\ {\ddot{E}}_{e\_(k)}\\ {\ddot{E}}_{e\_(k)} \end{array}\right]+Noise$$

#### Linear Reciprocal Model

The state estimator of the LR model-based KF is inspired by the agonist–antagonist EOM model of the human eye. The development of the LR model-based KF is detailed in [10]. The state-space representation of the corresponding process model, in the discrete form, can be written as

(15) $$\left[\begin{array}{c} \dot{X_{1}}\\ \dot{X_{2}}\\ \dot{X_{3}}\\ \dot{X_{4}} \end{array}\right]=\left[\begin{array}{cccc} 0 & 1 & 0 & 0\\ 0 & 0 & 1 & 0\\ 0 & 0 & 0 & 1\\ -C_{0} & -C_{1} & -C_{2} & -C_{3} \end{array}\right]\left[\begin{array}{c} X_{1}\\ X_{2}\\ X_{3}\\ X_{4} \end{array}\right]+\left[\begin{array}{c} 0\\ 0\\ 0\\ 1 \end{array}\right]\delta U+noise$$

where $X_{i}$ = *E*, $C_{i}$ are constants derived from the parameters that govern eye movements as detailed in [10].

#### Bandpass Filtering

The employed BP filter within the scope of this study is as mentioned in [1,9,16]. The FIR process involves the utilization of a bandpass filter with a bandwidth ranging from 0.5 to 35 Hz , a drift removal step, the incorporation of a notch filter set at 60 Hz , and the application of a Savitzky–Golay filter with a fifth-order polynomial and a frame length of 111.

#### Sensor Fusion

**Figure 2:**
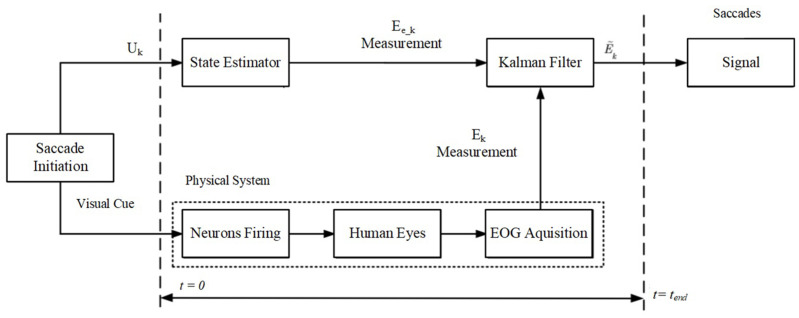
Schematic diagram of the model-based fusion algorithm. The saccades are initiated by visual cues leading to estimation and measurement. The velocity model is used to estimate the states of the eye and the Kalman filter is used to fuse the estimation with the measurement to generate the final output. To compare the velocity model with other model-based approaches, the state estimator was modified accordingly (e.g., with a constant acceleration model).

EOG dry electrodes are employed for the assessment of the corneal–retinal potential of the eyeball. The experimentally obtained signals are integrated with the saccade models delineated in this section. An overview of the comprehensive fusion algorithm is provided in Figure 2. The initiation of saccades is prompted by visual cues ( $U_{k}$ ), and their measurement is conducted via the raw EOG signals ( $E_{k}$ ) within the time interval from t=0 to $t=t_{end}$ . Subsequently, distinct state estimators inspired by the BM, CVM, CAM, and LR models are applied to compute $E_{e_{k}}$ and enhance the signal quality using the KF.

### 2.2. Experimental Description

#### 2.2.1. Participants

The primary objective of this human study is to assess the efficacy of the proposed denoising process in preserving EOG saccades by comparing them with saccades recorded by the EyeLink 1000, considered the gold standard in saccade measurement. This direct comparison of EOG and VOG saccades necessitates the simultaneous recording of saccades by both devices, as elaborated in Section 2.2.2. Procedure: In this experiment, any inherent variability in individual eye movements is mitigated, as both devices capture identical eye movements concurrently. This study involved thirteen healthy adults, aged 18 to 29 years, all of whom possessed normal or corrected visual acuity. The participants who regularly used eyeglasses or contact lenses to achieve 20/20 vision in their daily lives also wore these corrective lenses during the experiments. Ethical approval for conducting experiments on human subjects was granted by the University of British Columbia’s Behavioral Research Ethics Board (Approval No. H18-03792). Prior to participating in the study, written informed consent was obtained from all the participants. All the testing sessions took place at the UBC Vision Lab in Canada. The dataset reported in this study excluded data collected from participants with known eye disorders or individuals with significant optical corrections. However, two participants were excluded from the subsequent analysis and results: one participant did not meet the calibration requirements for pupil–corneal reflection (PCR) eye tracking, and another reported a diagnosis of Leber’s hereditary optic nephropathy, which is a congenital eye disorder associated with oculomotor abnormalities.

#### 2.2.2. Apparatus

**Figure 3:**
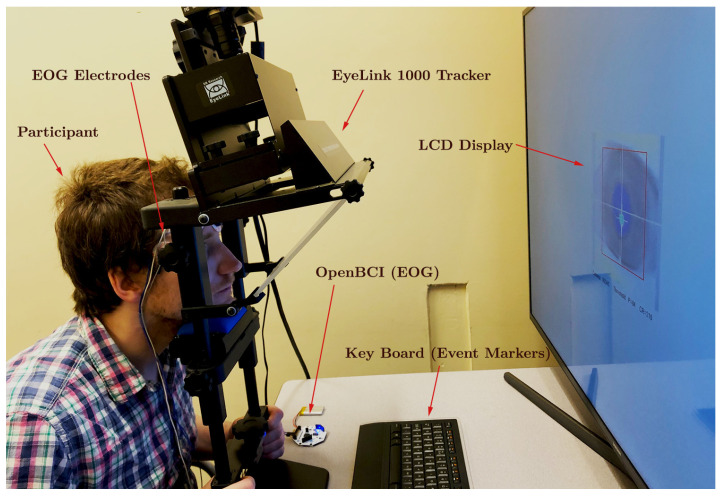
Experimentation arrangement of EyeLink 1000 tracker, OpenBCI device, and participant.

**Figure 4:**
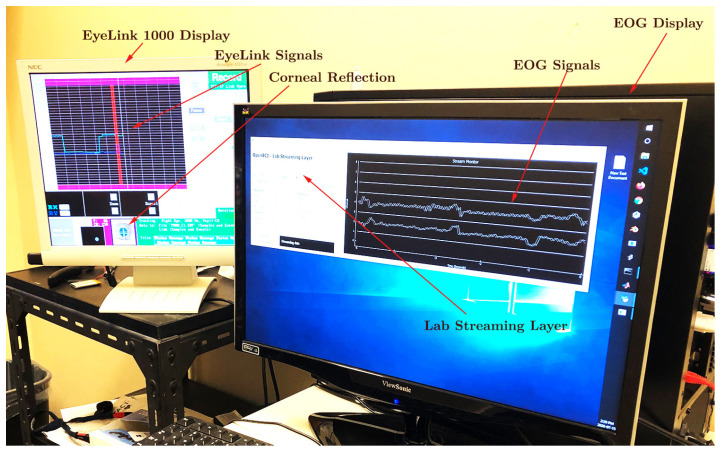
Recording of EyeLink 1000 tracker signals and OpenBCI EOG signals simultaneously using Lab Streaming Layer. The corneo-retinal potential is recorded by the electrodes placed on the outer canthus with respect to the electrode placed on the forehead. Horizontal saccades are directed to targets presented at −12, −11, 11, and 22 degrees of visual angle on the LCD screen.

To minimize any potential interference caused by head movements during data acquisition, the participants were instructed to maintain the utmost stillness by utilizing a chin-and-forehead rest. Monocular eye movement recordings were performed on the right eye at a sampling rate of 250 Hz , employing both the OpenBCI Cyton board for acquiring the EOG signal and the EyeLink 1000, resulting in the simultaneous generation of two parallel data streams. For EOG data collection, pre-gelled Skintact electrodes were securely positioned at the participant’s outer canthus and forehead. The EyeLink system tracked the gaze position based on the PCR, which was captured using its built-in infrared camera. Owing to the superior reliability of the EyeLink system, the data recorded by it were later employed for the calibration and validation of the EOG measurements (as illustrated in the experimental setup depicted in Figure 3). The stimulus presentation was under the precise control of the custom software developed using the PsychoPy3 package in Python. Visual cues were displayed on a 121.9 cm × 71.1 cm LCD monitor with a resolution of 1920 × 1080 pixels and a refresh rate of 30 Hz . Audio cues were emitted through standard computer speakers. A keyboard, placed in front of the participants, allowed them to initiate the next trial in the queue by pressing a key. The event data, including keyboard inputs, EOG signals, and EyeLink measurements, were synchronized in real time with a shared computer clock utilizing the Lab Streaming Layer (LSL) software. Although all the data samples were recorded using the same clock reference, some random time lags were observed in certain samples, as exemplified in Figure 4. These lags were unsystematic and sporadic, occurring only in the data collected from specific participants. These discrepancies were not considered in the qualitative analysis, as our calculations were independent of the temporal data provided by the time stamps.

#### 2.2.3. Procedure

The display featured a central marker in the form of a small circle, serving as the “home” fixation point. The magnitude of a saccade was quantified in relation to this home position, which was aligned with the participants’ midsagittal plane. Four target locations, labeled “A”, “B”, “C”, and “D”, were presented at angular deviations of −22° , −11° , 11°, and 22° from the visual angle, respectively. Notably, “A” and “B” targets were situated to the left of the home position, while “C” and “D” were positioned to the right. The investigation exclusively focused on horizontal saccades, and as such, all the target points were aligned along a horizontal axis traversing the center of the display. The initiation of each trial was contingent upon a keypress executed by the participant. Following trial initiation, an auditory cue was delivered, directing the participant to execute a saccade toward the designated target location. Subsequently, a second auditory cue, signified by a “ping” sound, prompted the participant to return their gaze to the home position, thus concluding the trial. The administration of the trials followed a pseudo-random order.

#### 2.2.4. Calibration

Before each testing session, a calibration procedure was conducted, comprising five consecutive trials for each of the four target locations, namely, A, B, C, and D. Subsequently, the raw EOG signal, measured in microvolts, was transformed into the eyeball angle through the application of linear parametric regression. Calibration points, denoted as $E_{peak_{a}verage}$ , were derived based on signal peak averages ( $E_{e_{peak}}$ ) and peak counts ( $N_{pc}$ ), following the formula:

(16) $$E_{\left(peak\_average\right)}\left(\theta \right)=\frac{1}{N_{pc}}\sum_{n=1}^{n=N_{pc}}\left[\min\max thresh\left(E\left[n\right]\right)\right]$$

#### 2.2.5. Kalman Filter

In KFs, a higher value of *Q* representing the model noise leads to an elevated gain and imparts greater significance to the measurement, albeit at the cost of introducing a time lag. Conversely, a lower *Q* value enhances the accuracy but may result in a delayed response. Therefore, the selection of an appropriate *Q* value necessitates a trade-off between the time response and accuracy, as discussed in references [10,26,37]. In this study, the *Q* values were determined using an iterative trial-and-error methodology. The values of *R* (representing sensor noise) were computed based on the mean of the standard deviations of the normalized errors observed in the calibration trial data.

#### 2.2.6. Study Parameters

Throughout the data processing phase, the trials were delineated by recorded event markers, which corresponded to audio cues and keyboard inputs denoting the commencement and conclusion of each trial. The saccades were subsequently identified by detecting the two change points employing MATLAB’s “findchangepts” function, a tool designed to pinpoint abrupt shifts within a signal. The peaks situated between these two change points were extracted using MATLAB’s “findpeaks” function, and their average values were employed to define the saccade amplitude, corresponding to the angular displacement of the eyeball for both the EOG ( $\theta_{EOG}$ ) and EyeLink ( $\theta_{EL}$ ) data. An additional key feature calculated during the analysis was eye movement latency, denoted as $t_{lat_{EOG}}$ and $t_{lat_{EL}}$ , representing the time interval between the audio cue signaling the commencement of the trial and the first identified change point. Numerical differentiation was applied to the EOG and EL signals to derive the peak velocities, defined as $V_{EOG}$ and $V_{EL}$ , denoting the maximum slope of the saccade response.

Furthermore, two definitions of error were considered: the EOG error relative to EL ( $E_{EOG-EL}=\left|\theta_{EOG}-\theta_{EL}\right|$ ) and the measurement error (absolute error in EOG or EL, $E_{EOG/EL}=\left|\theta_{EOG/EL}-\theta \right|$ , where θ denotes the physical angle in the experimental setup). Additionally, for both EL and EOG, the accuracy and precision were assessed by examining the mean value and variance of the percentage of the normalized error (measurement error or absolute error, expressed as a percentage of the normalized error). To compare the noise power and signal power of the filtered signals, the signal-to-noise ratio (SNR) was employed. In this context, the SNR was defined as follows:

(17) $$SNR_{\left(dB\right)}=10\log[\frac{\sum_{n=1}^{n=k}\bar{E_{k}}}{\sum_{n=1}^{n=k}\left[E_{k}-E_{k}^{\prime }\right]]}$$

Furthermore, the analysis involves the consideration of the computation time ( $t_{c}$ ) and nonlinear curve fitting of the peak velocity, utilizing Equation (5). A comprehensive overview of the parameters employed in this study is provided in Table 1.

## 3. Results and Discussion

**Figure 5:**
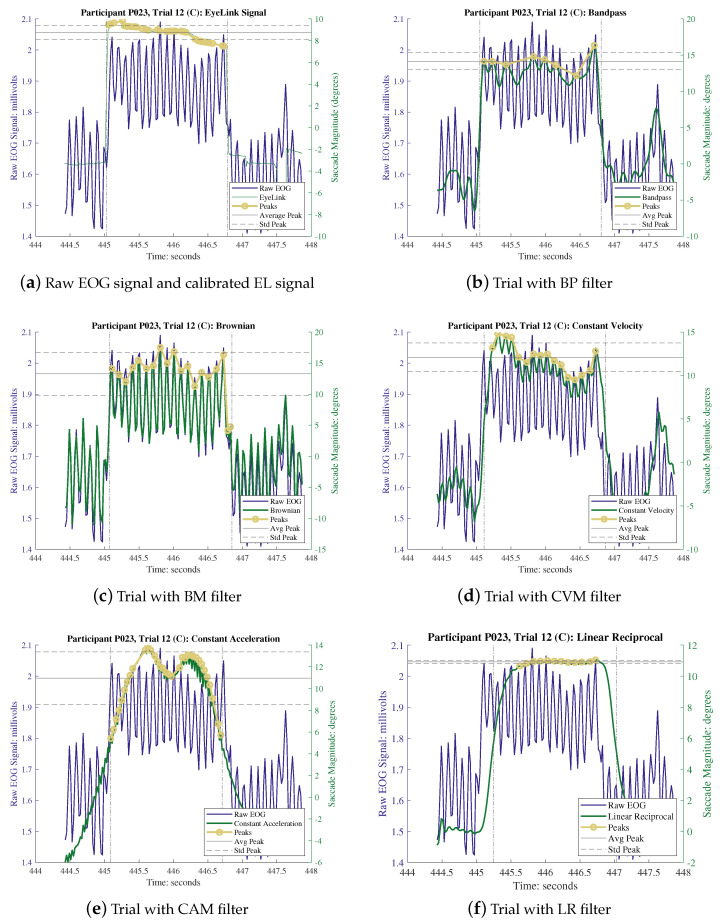
The responses of different filters, when applying on raw EOG signals. The comparison against (**a**), raw EOG signal and EL signal demonstrate the contribution of each filter. Note: in (**a**), left Y axis and blue line represent the raw EOG signal and right green axis represents the EL signal. From (**b**) to (**f**), the left Y axis and blue line are the same and the right green axis represents the filtered signal.

The proper calibration of the KF is of utmost importance for optimizing the performance of each of these filters. The determination of the *R* values was carried out based on the results of the individual calibration trials, while the *Q* values remained consistent across various methods, with *Q* being determined using a trial-and-error approach as elaborated in Section 2.2.5. The values of both *R* and *Q* employed in each trial are documented in Table 2. Each experimental session consisted of two distinct types of trials, wherein the participants first engaged in a series of calibration trials followed by a sequence of experimental trials. The data recorded during the calibration trials were employed in conjunction with linear regression techniques to calibrate the subsequent experimental trials. The calibration factors generated through this process are presented in Table 3. It is worth noting that both the EOG and EL recordings underwent the same calibration process. However, it is important to emphasize that the recorded EL data, due to its inherent high quality, did not require denoising and were considered as the ground truth for this experiment. To process the recorded signals, a polynomial piece-wise detrend function in MATLAB was employed. This function was utilized to rectify any drift between the trial markers, specifically the “start” and “end” events, and to normalize the isolated EOG saccades to their baseline value, effectively removing the systematic baseline drift. Outliers within the data were identified and removed using the interquartile range method, which entails removing data points that fall outside a range of 1.5 times the interquartile range from the first and third quartiles, thereby ensuring a dataset devoid of bias. The application of different denoising methods yielded varying results in terms of signal denoising and the reduction in outliers in the EOG saccades. Table 2 provides a summary of the percentage of outliers removed after each denoising method was applied (namely, 13.48% for BP, 10.90% for BM, 12.05% for CVM, 12.02% for CAM, and 12.2% for LR). On average, all the model-based filters exhibited a 12.5% improvement in comparison to the bandpass filter method. Figure 5 illustrates an example of the recorded data extracted between the “start” and “end” event markers for participant P023 during the execution of a C trial (i.e., a saccade of $11^{{}^{\circ}}$ ). Subfigure (a) displays the raw recorded signal (comprising both the EOG and EL data), while subfigures (b) to (e) depict the outcomes following the application of various denoising methods. A thorough examination of these figures reveals that CVM has the capability to preserve EOG saccades more effectively when compared to other filters, as the overall signal shape closely resembles the EL records. In order to further assess and quantify the performance of the CVM, a comprehensive analysis was conducted, encompassing a correlation study, a numerical feature analysis of the EOG parameters, and an error analysis, utilizing data from all the participants.

Given the utilization of two distinct recording devices in this study, an examination was conducted to assess the feature correlation between different features and the applied denoising methods, thereby establishing a relationship between each filter and the features derived from the two devices. Specifically, the θ , $E_{EOG/EL}$ , $V_{p}$ , and $t_{s}$ of EOG and EL were computed for each denoising method and are comprehensively presented in Table 4. Notably, all these methods exhibited a robust correlation, with the KF-based filter techniques demonstrating an enhanced correlation of approximately 0.8% in amplitude. It is pertinent to note that θ , representing the average peak values of the saccade at fixation, is a key feature influencing the shape of the signal. Furthermore, the SNRs were computed and their results are displayed in Table 5 to affirm the efficacy of all the employed denoising methods in generating a potent output signal. As anticipated, the average $t_{c}$ (also showcased in Table 5) was higher for the model-based KF in comparison to the BP method, aligning with expectations.

**Figure 6:**
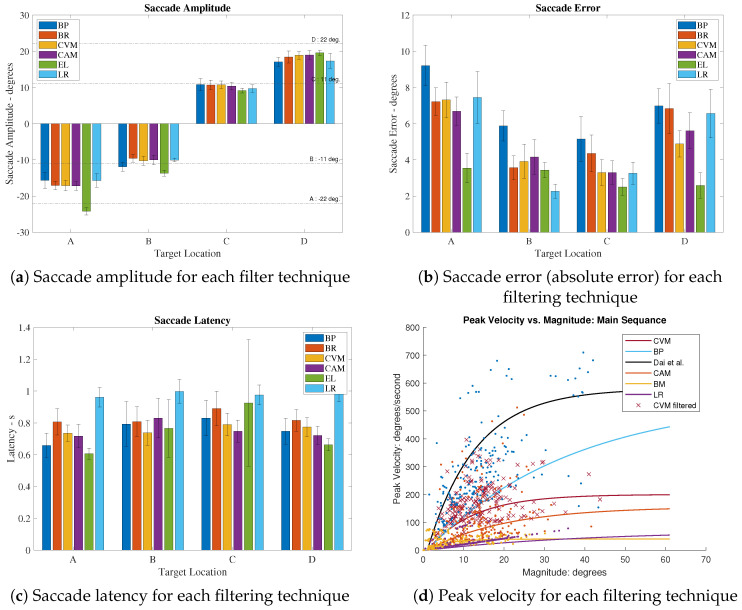
Extracted saccade characteristics from EyeLink and filtered EOG signals, grouped by target locations A, B, C, and D, averaged for all participants; Dai et al. [32].

To conduct a more comprehensive examination of the capacity to retain EOG saccades within the proposed denoising procedure, four key parameters were subjected to analysis. Figure 6 provides a visualization of the average values of these primary parameters, considering data from all the participants (encompassing both EOG and EL saccades) throughout the study, subsequent to the application of various denoising methods. In Figure 6a,b, the behavior of θ following the application of each denoising method is depicted. As summarized in Table 6, the utilization of the CVM has demonstrated a notable enhancement of the EOG signal by 28.7% in comparison to the BP method, resulting in a reduction in the $E_{EOG}$ . Furthermore, when compared to the EL signal, the overall EOG signal has experienced a 22.3% improvement ( $E_{EOG-EL}$ ). Figure 6c illustrates the $t_{s}$ of the saccades, revealing that the application of these denoising methods does not have a significant impact on the EOG saccade $t_{s}$ . In Figure 6d, the relationship between the peak velocity ( $V_{p}$ ) and magnitude after the application of various denoising methods is elucidated. The filtered saccades were fitted to Equation (5) and compared with the data from reference [32]. The resulting fitted curves produced values of 62.13 for CVM, 110.63 for BP, 83.86 for CAM, 18.78 for BM, and 16.11 for LR, all fitted with p<0.001 . Although BM and LR exhibited low RMSE values, their $V_{p}$ was diminished post-filtering. In contrast, the CVM, BP, and CAM yielded far more realistic $V_{p}$ values compared to the peak velocity versus amplitude data published in reference [32]. Among these methods, CVM displayed the lowest RMSE, indicating the closest fit to the $V_{p}$ formula. This analysis underscores that the CVM filter has not only effectively denoised the signal but has also preserved the EOG saccades during the denoising process. Representative examples of randomly selected trials are provided in Appendix A for further illustration.

**Figure 7:**
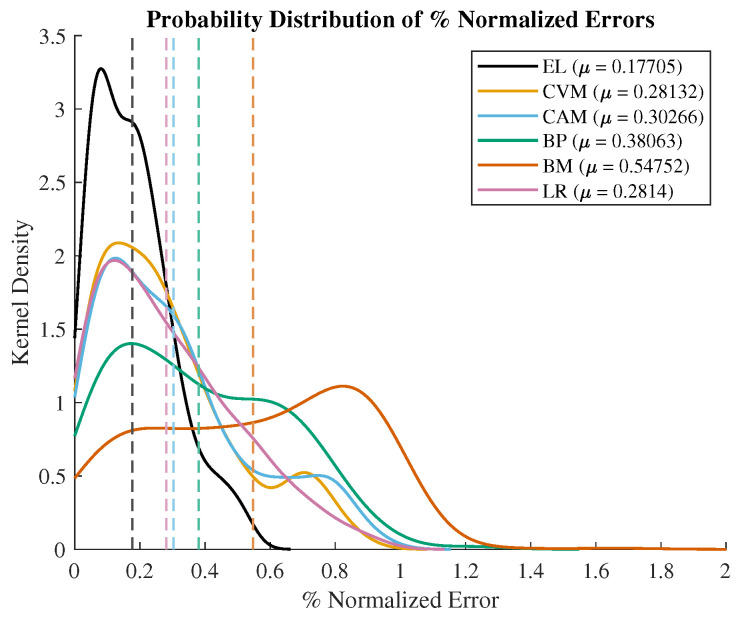
Probabilistic density distribution (kernel density) of % normalized error. Mean values marked as dash lines.

To conduct a more in-depth performance analysis of the CVM in comparison to other denoising methods, we examined the kernel density of the probability distribution of the percentage of the normalized error, as depicted in Figure 7. This was computed by evaluating the (measured value − true value)/true value after the removal of outliers using 1.5 times the IQR criterion. The calculated mean values ( μ : EL = 0.18, CVM = 0.28, CAM = 0.3, BP = 0.38, and BM = 0.55) of this distribution serve as an indicator of the accuracy of each technique concerning EL. It is evident from the analysis that the CVM exhibits an improvement in accuracy of 2%, 10%, and 27% when compared to the CAM, BP, and Brownian techniques relative to EL. Furthermore, two-sample *t*-tests were conducted against EL, and all the filtering techniques demonstrated the rejection of the null hypothesis, thereby establishing the significance of the difference between the recorded percentages of the normalized error values (*p* values: CVM— $1.65\times 10^{-6}$ , CAM— $1.59\times 10^{-7}$ , BP— $3.94\times 10^{-16}$ , and BM— $2.15\times 10^{-54}$ . Consequently, the CVM showcases superior accuracy in comparison to other techniques concerning EL. The variance ( $\sigma^{2}$ : EL = 0.03, CVM = 0.06, CAM = 0.07, BP = 0.08, and Brownian = 0.08) of the percentages of the normalized error values also reveals that the CVM demonstrates a 1%, 2%, and 2% enhancement in precision relative to the CAM, BP, and Brownian techniques concerning EL.

## 4. Conclusions

In this paper, we introduce an adaptive filtering method grounded in the assumption of constant velocity, which entails considering the rate of change in the corneal–retinal potential as constant. We then conduct a comparative assessment of this CVM against established techniques, such as BP, BM, CAM, and LR-based methods. To rigorously evaluate these methods, we employ controlled experiments wherein saccades are concurrently measured using an OpenBCI Cyton Biosensing board (for EOG signal acquisition) and an EyeLink 1000 eye-tracker. We extract essential parameters including θ , $E_{EOG/EL}$ , $V_{p}$ , and $t_{s}$ from the signals and employ them to assess the performance of each denoising method, with particular emphasis on characterizing the efficacy of the recently introduced CVM approach. The CVM exhibited the most superior performance, manifesting in the filtered data by achieving the lowest errors ( $E_{abs_{EOG}}=$ 9.52° and $E_{EOG-EL}=$ 10.63° ). The outcomes reveal that, in comparison to the physical size of the saccade, the CVM enhanced the EOG signal by approximately 29%, and in relation to the EyeLink (EL) recordings, it improved it by over 22% when contrasted with the BP filter. Furthermore, compared to the physical size of the saccade, the CVM outperformed the BP filter by approximately 2% and surpassed it by over 21% when evaluated against the EL recordings. The kernel density distribution of the normalized error percentages indicates that the introduction of sound mathematical models can augment the filtering capabilities of Kalman filters (KFs). As demonstrated by the results, both the CVM and CAM substantially elevated the accuracy and precision of the eye movement recordings. Specifically, the CVM exhibited an average improvement of 13% in accuracy and 3% in precision when compared to other denoising methods.

In Figure 7, we present the computed means derived from the entire dataset, characterizing the four key saccade features: amplitude (Figure 6a), error (Figure 6b), peak velocity (Figure 6c), and latency (Figure 6d). A comparative analysis is performed between the means obtained from the EyeLink and the EOG signals, utilizing various filtering methods, including the BP, Brownian, CAM, and CVM. The accompanying error bars depict the standard deviation. It is important to note that the EyeLink signal is regarded as the primary reference point for comparison, given its recognized accuracy in measurements. Upon examining Figure 6a,b, a discernible pattern emerges, wherein saccades with smaller amplitudes exhibit superior accuracy in the EOG signal when compared to saccades with relatively larger amplitudes. Specifically, targets A and D correspond to the most peripheral goal locations, entailing saccade amplitudes of $\pm 22^{\circ }$ concerning the home position, where the induced charge approximates zero. This peripheral positioning may introduce nonlinearity into the signal, potentially diminishing the accuracy of the recording. A similar trend is observed in Figure 6b, where the saccades directed toward targets B and C display reduced errors in comparison to those aimed at targets A and D. It is noteworthy that the application of the filtering methods has a negligible impact on the latency of the signal. This outcome aligns with expectations, as these filters are not anticipated to influence the temporal resolution of the signal.

The CVM-based KF employed fixed values for *Q* and R″ , as outlined in Section 2.2.5. These values were established at the outset of each session and remained unaltered throughout the signal processing phase. It is plausible that this static configuration may have constrained the algorithm’s overall performance. Thus, it is posited that the implementation of a methodology to dynamically compute real-time adjustments for the *Q* and *R* values could enhance the efficacy of the signal filtering process. As indicated in Figure 6d, saccades demonstrate a constant velocity behavior for only high degrees and speeds. Consequently, there may be a necessity to modify this method for effective application in very short range saccades and low-speed scenarios, such as those encountered in smooth pursuit eye movements. Furthermore, it is important to note that the method does not encapsulate any mechanical or electrical characteristics of the eyeball or eye muscles, unlike models such as LR which incorporate electromechanical properties. Therefore, in instances of extreme conditions, such as individuals with disorders affecting the eyeball or eye muscles, the method, in its current state, may encounter challenges in accurately enhancing signals. Furthermore, the amalgamation of sensor data from both EyeLink (EL) and EOG sources has the potential to yield highly accurate EOG saccades. However, such an approach may impose limitations on the practical applicability of this technology, particularly in scenarios requiring the detection of closed-eye movements. One notable limitation in the present study pertains to the collection of EOG data under conditions of consistent illumination. Given the sensitivity of the corneal–retinal potential to variations in lighting conditions, this factor assumes significance in real-world applications where illumination levels are subject to change. Future research endeavors could extend upon this foundation by exploring the filtering capabilities of this algorithm when applied to EOG records acquired under varying lighting conditions. Additionally, there exists the opportunity to investigate novel machine learning-based techniques for EOG denoising that offer comparable capabilities. The current investigation is specifically concentrated on saccadic eye movements, given their prevalence and frequent occurrence in daily human activities. Subsequent research endeavors are anticipated to extend the scope of these methods to encompass less common eye movement types, such as smooth pursuit movements and vestibular–ocular reflexes.

## Figures and Tables

**Table 1:** Definitions of study parameters.

Study Parameter	Symbol	Definition
Amplitude	θ	Angle of the landing position of eye from the center
Peak velocity	$V_{p}$	Average of the maximum peaks at the fixation
Latency	$t_{s}$	Time taken to initiate the saccade after receiving cue
Abs. relative error	$E_{EOG-EL}$	Absolute of difference between EOG and EL mag.
Abs. EyeLink error	$E_{EL}$	Absolute difference between EL and physical mag.
Abs. relative error EOG	$E_{EOG}$	Absolute of difference between EOG and physical mag.
Normalized error	$E_{N}$	Normalized absolute relative error
Computation time	$t_{c}$	Time taken to process the data between markers
Signal-to-noise ratio	SNR	Ratio of the signal power to the noise power

**Table 2:** Percentage of outliers (Out.%) excluded, *Q*, and *R* values for each participant.

	BP	BM		CVM			CAM			LR *	
**PID**	**Out. %**	**Out. %**	**Q**	**Out. %**	**Q**	**Out. %**	**Q**	**R**	**Q**	**R**	**Out. %**
P20	14.08	10.06	0.01	8.66	0.5	11.57	0.5	10.53	0.5	11	13
P22	9.95	7.52	0.01	8.76	0.5	7.19	0.5	15.56	0.5	16	11.1
P23	15.25	15.16	0.01	15.70	0.5	16.19	0.5	8.06	0.5	8	12.8
P24	6.31	8.29	0.01	4.71	0.5	6.58	0.5	15.55	0.5	16	7.3
P25	14.13	17.88	0.01	17.49	0.5	13.59	0.5	16.64	0.5	17	18
P26	21.24	9.26	0.01	8.50	0.5	11.09	0.5	5.55	0.5	6	10
P27	12.52	11.54	0.01	9.84	0.5	9.15	0.5	150.40	0.5	150	13.9
P28	16.03	14.15	0.01	14.49	0.5	14.51	0.5	10.14	0.5	10	14.8
P29	14.08	11.37	0.01	15.72	0.5	14.42	0.5	21.71	0.5	22	12.1
P32	17.10	19.24	0.01	19.20	0.5	17.43	0.5	22.85	0.5	13	15
P33	7.63	6.88	0.01	9.42	0.5	10.49	0.5	173.31	0.5	173	6.4

*Note:* Data points were excluded as outliers if they fell outside the interquartile range at the participant level. BP = bandpass, BM = Brownian motion model, EL = EyeLink 1000, CAM = constant acceleration model, CVM = constant velocity model, PID = Participant ID. * The results for LR model were extracted from [10].

**Table 3:** Calibration factors for EOG based on each filtering technique ( $mV\deg^{-1}$ ) and EL (px $\deg^{-1}$ ).

PID	BP	BM	CAM	LR *	EL	CVM
P20	0.25	0.28	0.29	0.03	0.07	0.25
P22	0.21	0.23	0.20	0.01	0.08	0.18
P23	0.48	0.40	0.52	0.06	0.07	0.47
P24	0.29	0.47	0.41	0.02	0.07	0.31
P25	0.12	0.08	0.09	0.03	0.08	0.09
P26	0.15	0.14	0.49	0.03	0.07	0.36
P27	0.50	0.99	0.57	0.04	0.07	0.42
P28	0.23	0.24	0.30	0.04	0.07	0.27
P29	0.27	1.11	0.42	0.02	0.08	0.37
P32	0.20	0.19	0.26	0.03	0.07	0.24
P33	0.17	0.37	0.25	0.02	0.08	0.25

*Note:* PID = Participant ID. * The results for LR model were extracted from [10].

**Table 4:** Feature correlations between the filtered EOG signal and EL.

Feature	BP	BM	CAM	LR *	CVM
Amplitude	0.991	0.999	0.999	0.997	0.999
Error	0.654	0.139	0.540	0.732	0.671
Peak Velocity	0.976	0.935	0.157	0.485	0.967
Latency	0.999	0.649	0.576	0.336	0.583
Improvement (%)	–	8	8	7.5	8

*Note:* Correlations were computed using Pearson’s method. * The results for LR model were extracted from [10].

**Table 5:** Signal-to-noise ratio and computation time.

Measurement	BP	BM	CAM	LR	CVM
SNR	10.72	12.2	11.46	9.01	11.48
$t_{c}$	0.48	0.22	0.94	0.72	0.98
% improvement in $t_{c}$ with respect to BP	−	−13.47	95.36	49.19	104.43

*Note:* BM has a lower $t_{c}$ and other methods have a higher $t_{c}$ than BP.

**Table 6:** Mean errors (DVA) and improvement (%) with respect to bandpass for model-based KFs.

Parameters		BP	BM	CAM	CVM	LR *
** $E_{\_abs\_EL}$ **	Mean	2.85	2.85	2.85	2.85	2.85
	SD	0.42	0.42	0.42	0.42	0.42
$E_{\_abs\_EOG}$	Mean	6.80	5.49	4.93	4.85	6.75
	SD	1.04	0.95	0.85	0.84	1.23
	Improv. %	-	19.30	27.50	28.70	1
$E_{\_EOG\_EL}$	Mean	7.57	6.76	6.18	5.88	7.83
	SD Improv. %	1.13	1.16	0.97	0.93	1.28
	Improv. %	-	10.70	18.40	22.30	3.43

*Note:* * The results for LR model were extracted from [10] and $E_{\_abs\_EL}$ mean and SD remain unchanged, as the filters were exclusively applied to EOG signals, with EL considered as the gold standard.

**Figure A1:**
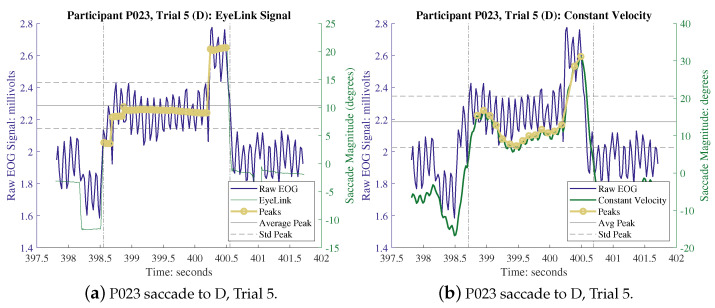

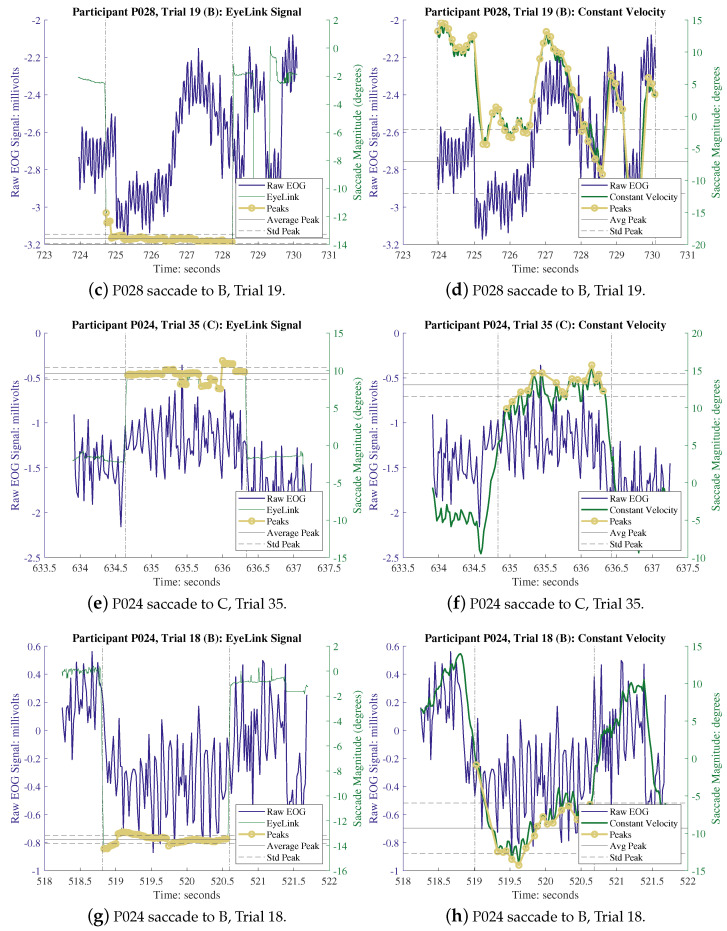
Randomly selected trials. (**a**,**c**,**e**,**g**) are EL vs. raw EOG and (**b**,**d**,**f**,**h**) are raw EOG vs. CVM.

## Data Availability

The code and data can be downloaded from the following source: https://github.com/HiroshanOU/EOG_CVM (accessed on 30 December 2023).
